# Advancing inclusive healthcare through PBPK modelling: predicting the impact of CYP genotypes and enzyme ontogenies on infant exposures of venlafaxine and its active metabolite O-desmethylvenlafaxine in lactation

**DOI:** 10.1007/s10928-025-09969-4

**Published:** 2025-04-04

**Authors:** Xian Pan, Karen Rowland Yeo

**Affiliations:** 1 Certara UK Limited, Certara Predictive Technologies Division, Sheffield, UK; 2 Certara UK Limited, Level 2-Acero, 1 Concourse Way, Sheffield, S1 2BJ UK

**Keywords:** Anti-depressant, Lactation, PBPK, Venlafaxine, O-desmethylvenlafaxine

## Abstract

About 15–20% of women experience postnatal depression and may seek advice about medication use whilst breastfeeding. Venlafaxine is a potent and selective neuronal serotonin-norepinephrine reuptake inhibitor indicated for treating major depressive disorders. The drug is mainly metabolised by cytochrome P450 2D6 (CYP2D6) to its active metabolite O-desmethylvenlafaxine (ODV), with small contributions from CYP2C9 and CYP2C19. Subsequently, the formed ODV undergoes CYP3A4- and UGT-mediated metabolism and renal excretion. A physiologically based pharmacokinetic (PBPK) model describing the disposition of both venlafaxine and ODV was developed. Consistent with observed data, simulations showed that exposure of the combined active moieties (venlafaxine plus ODV) was similar for both CYP2D6 extensive (EM) and poor metaboliser (PM) subjects. Clinical lactation data for venlafaxine were available from several studies but CYP genotypes were not recorded. Interestingly, based on simulated exposures in breast milk, the estimated average relative infant daily dose (RIDD) ranged from 3.8% for all EMs to 7.6% for all PMs of CYP2D6, CYP2C9 and CYP2C19. Furthermore, simulations in breastfed infants indicated that both CYP polymorphisms and enzyme ontogenies contribute to the significant variability that is observed clinically but the combined exposures of venlafaxine and ODV remain below the thresholds that have been reported for adverse events in adults and children. The data generated here add to the existing knowledge base and can help clinicians and their patients make a more informed decision on the use of venlafaxine during breastfeeding.

**Supplementary Information:**

The online version contains supplementary material available at 10.1007/s10928-025-09969-4.

## Introduction

Depression and anxiety are common in the postnatal period, affecting around 15–20% of women [1]. The prevalence and duration of breastfeeding is increasing as it continues to be recognised as the optimal source of infant nutrition [2, 3]. Thus, over the coming years, it is likely that more women will be seeking advice from healthcare professionals about the use of antidepressant medication during lactation. Current guidelines on treatment decisions recommend that clinicians make an individualised assessment and weigh the risk of exposing the infant to the maternal medication against the benefits of breastfeeding [1].

Venlafaxine is a potent and selective neuronal serotonin-norepinephrine reuptake inhibitor indicated for treating major depressive disorders. Both the parent drug and its metabolite O-desmethylvenlafaxine (ODV) demonstrate similar antidepressant efficacy [4, 5]; thus, evaluating the total concentration of both active moieties is essential. Venlafaxine is primarily metabolised via cytochrome P450 2D6 (CYP2D6), with minor contributions from CYP2C9 and CYP2C19, leading to the formation of ODV [6]. Subsequently, the formed ODV undergoes CYP- (primarily CYP3A4) and UGT-mediated metabolism and renal excretion [7, 8]. Genetic polymorphisms in CYP2D6, CYP2C9, and CYP2C19 can influence venlafaxine metabolism and ODV formation, and hence exposure of both active components. Common side effects of venlafaxine treatment include nausea, headaches, dizziness and constipation. However, the concurrent side effects have rarely been reported in infants exposed to venlafaxine and ODV through breastfeeding according to lactation database [9]. Additionally, venlafaxine use during breastfeeding has been suggested to alleviate neonatal abstinence symptoms, as indicated in a case report [10].

The US FDA guidance on the conduct of clinical lactation studies issued originally in 2005 and updated in 2019 [11], recommends that the milk to plasma ratio (M/P), estimated infant daily dose (IDD) and relative infant daily dose (RIDD) be reported. Furthermore, it indicates that to be able to fully assess the potential of a drug to clinically affect a breastfed infant, concentration time profiles of the drug in breast milk and maternal plasma (and ideally the infant) are required. Clinical lactation data indicate that both venlafaxine and formed ODV are excreted into breast milk with mean M/P ratios ranging from 2 to 3 [12, 13]. The combined (venlafaxine plus ODV) mean estimates of RIDD ranged from 6.4% [12] to 8.1% [13], falling below the 10% nominal cutoff which is often cited for safe medication use in lactating women [14]. As with other drugs, there has been much debate about how these parameters (i.e. the M/P ratio, the RIDD, infant plasma concentrations and reported adverse events) can be used to inform the safe use of venlafaxine. Both the FDA and EMA recommend that when prescribing venlafaxine to lactating mothers, the potential risks to the infant, the need for treatment, and the benefits of breastfeeding should be considered [11, 15]. Despite the availability of clinical lactation data, there is no specific guidance to help clinicians, and their patients make a more informed choice regarding the risks and benefits of using venlafaxine during breastfeeding. Expanding the existing knowledge on the use of venlafaxine in breastfeeding women is key to advancing this area. Given the potential impact of CYP2D6, CYP2C9 and CYP2C19 genotypes on venlafaxine disposition as well as the various ontogenies associated with these enzymes, it is necessary to explore key factors that may contribute to the interindividual variability observed in maternal (M/P ratios and RIDD) and infant exposures of venlafaxine and ODV [12, 13].

**Fig. 1:**
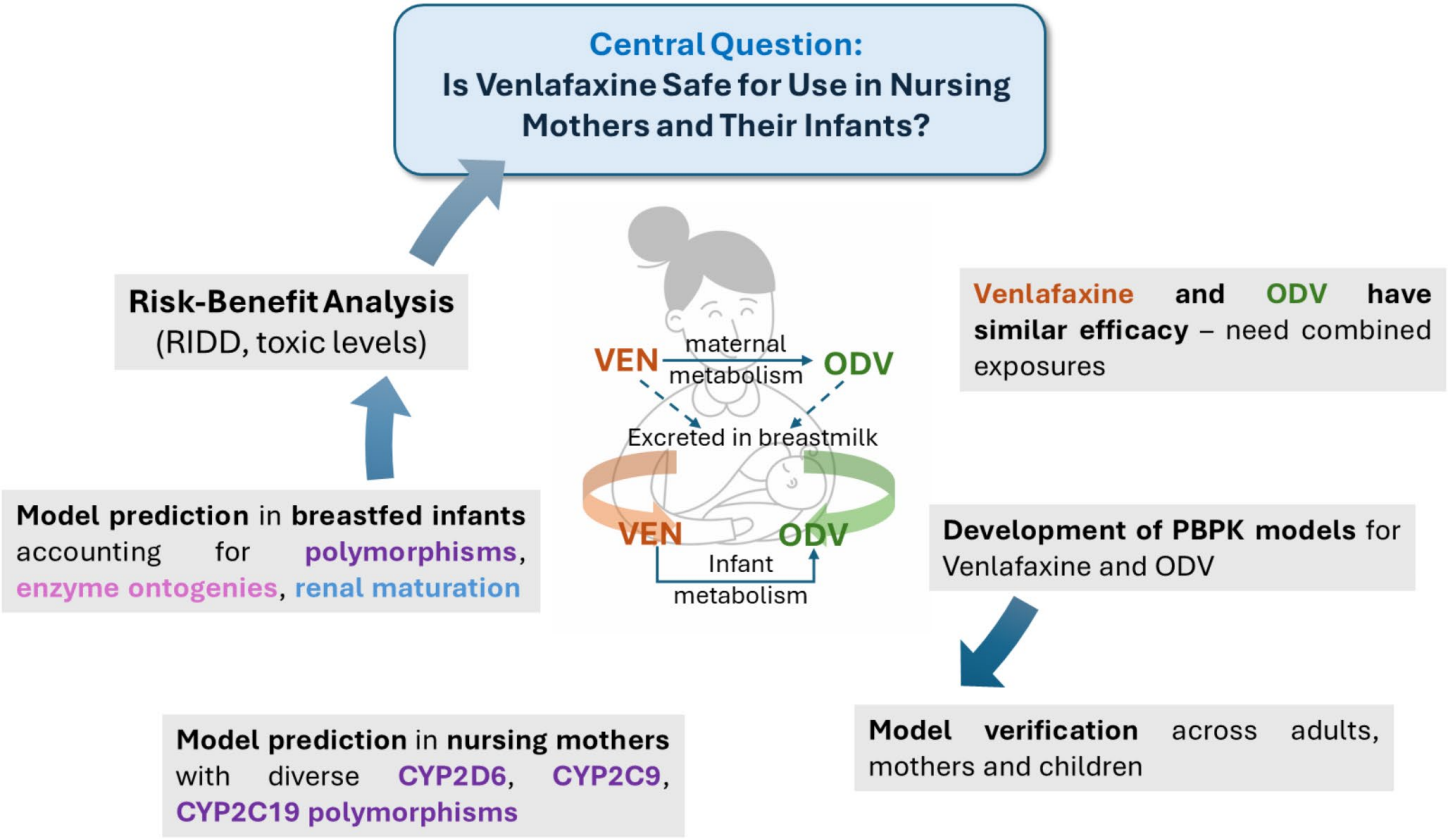
Schematic of workflow

Models to predict M/P ratios from the physicochemical properties of a drug are available and can be integrated within a physiologically based pharmacokinetic (PBPK) to simulate plasma concentration-time profiles of venlafaxine in mothers and breastfeeding infants [16]. Of note, PBPK models consider the time-variant complex interplay between physiological parameters and drug-related characteristics in nursing mothers and infants and can be used to simulate scenarios where clinical data are lacking or sparse. A PBPK model was developed previously to investigate the drug-gene interactions (DGIs) of venlafaxine and ODV according to CYP2D6 and CYP2C19 polymorphisms [17]. The authors implemented CYP2D6 and CYP2C19 activity score-dependent metabolism to simulate exposures for various DGIs which were then verified using clinical data involving subjects with the various polymorphisms. Herein, we describe the development and verification of an alternative PBPK model for venlafaxine and formed ODV, with complex absorption that considers venlafaxine immediate release (IR) and extended release (ER) formulations in mothers, as well as the intake and absorption of venlafaxine and ODV from breast milk in infants. Drug-drug interactions with inhibitors of CYP2D6, CYP2C19, CYP2C9 and CYP3A4, which were used to verify the relative contributions of these CYP enzymes in the elimination of venlafaxine and ODV, are also detailed. The primary objective of this research was to complement the extant clinical lactation data by applying the verified PBPK model to assess the impact of CYP2D6, CYP2C9, and CYP2C19 genetic polymorphisms and associated ontogenies on exposures of both active moieties in maternal and infant plasma during breastfeeding and elucidate key contributors to the associated interindividual variability (Fig. 1).

## Methods

### Software

The Simcyp (version 21) population-based PBPK simulator (Simcyp, Sheffield, UK) was used to develop venlafaxine and ODV PBPK models and perform all the simulations. The PBPK models for quinidine, ketoconazole, and cimetidine used for model verification against clinical DDI studies were published previously [18–20]. Clinical study data from the literature were digitised with GetData Graph Digitizer version 2.22.

### Venlafaxine and ODV PBPK models

**Fig. 2:**
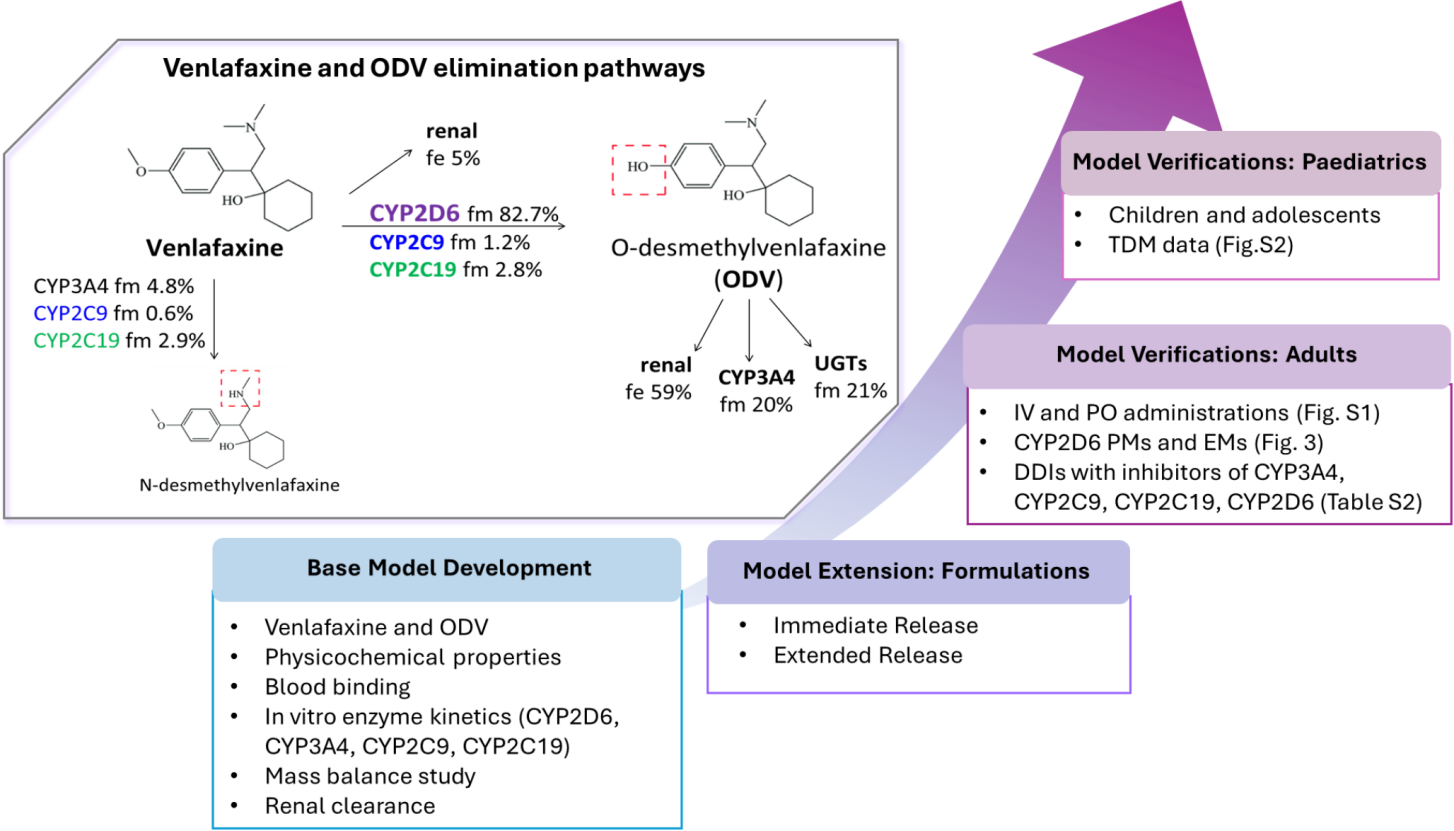
Flow chart of the development and verification venlafaxine and ODV PBPK models. PM: poor metaboliser; EM: extensive metaboliser; TDM: therapeutic drug monitor; fm: fraction metabolised by CYP/UGT enzymes; fe: fraction excreted renally

The workflow for the PBPK model development and verification of venlafaxine and ODV is described in Fig. 2. Venlafaxine primarily undergoes metabolism via CYP2D6, with lesser contributions from CYP2C9 and CYP2C19, resulting in the formation of its active metabolite, ODV. The latter is further metabolised primarily by CYP3A4, undergoes UGT-mediated conjugation and is excreted renally. The elimination pathways for venlafaxine and ODV, including the estimated fraction metabolised by CYP enzymes (fm) and fraction excreted renally (fe), are illustrated in Fig. 2. Several UGT isoforms including UGT1A1, 1A3, 2B4, 2B15, 2B17 contribute to ODV O-glucuronide formation, but the exact contribution of each UGT isoform is unknown [8]. Thus, UGT-mediated elimination of ODV is represented by an additional clearance in human liver microsomes in our model. Distribution for both venlafaxine and ODV was described by the whole body full PBPK distribution model. The advance dissolution, absorption and metabolism (ADAM) model [21] was used to describe the absorption of venlafaxine IR and ER formulations, as well as the intake of venlafaxine and ODV from breast milk. The input parameters of venlafaxine and ODV PBPK models are detailed in Table S1.

### Clinical data

Individual PK data for breastfeeding mothers and their infants from two clinical studies were used.

(i) Study 1: PK data were reported for two out of three mothers aged 26 and 35 years, who were on stable twice-daily (BID) doses of venlafaxine IR (150 mg BID and 225 mg BID) for 0.5 and 5 months respectively. Their infants, aged 0.37 and 1.3 months (38 weeks at delivery), were breast-fed according to their normal pattern during the study. Venlafaxine concentrations were not detectable in all infant blood samples [22].
(ii) Six breastfeeding women aged 30–41 years and their infants, including one pair of twins, aged 2.7 to 10.3 months participated [12]. The individual age of the mothers and their infants were not reported. Mother subjects 1–5 received venlafaxine IR (Subject 1 [likely a CYP2D6 PM], 112.5 mg BID; Subject 2, 150 mg BID; Subjects 3 and 4, 150 mg in the morning and 112.5 mg at night; Subject 5, 150 mg in the morning and 75 mg at night), while Subject 6 received 225 mg venlafaxine ER once per day in the morning. Infants were breastfed 5–6 times daily. Infants of mother Subjects 2 and 6 received supplementary solid food 2–3 times per day. Infant of mother Subject 3 received supplementary formula milk (50–120 mL per feed). Maternal PK profiles of both venlafaxine and ODV were reported. Infant blood samples were collected at 6.2 to 6.8 h (average 6.5 h) post maternal dose. Venlafaxine concentrations were undetectable in two infants.

In addition, PK data from healthy adult subjects were collated from 3 clinical studies to verify the model, including single intravenous (IV) dose of venlafaxine and desvenlafaxine (ODV), as well as a single oral dose of venlafaxine IR and ER [23–25]. Clinical DDI studies, including those with quinidine (CYP2D6 inhibitor), ketoconazole (CYP3A4, CYP2C9 and CYP2C19 inhibitor), and cimetidine (CYP2D6 inhibitor) [26–28] were collated, to verify each CYP component of the venlafaxine and ODV models. Furthermore, therapeutic drug monitoring (TDM) data from one clinical study involving children and adolescents and one retrospective analysis that included adolescents were utilised [29, 30]. The details of these clinical studies are provided in the supplementary materials.

### Virtual populations

Simulations were performed using virtual populations from the Simcyp population library, including North European healthy adult and paediatric populations. The demographic, physiological, and biochemical parameters used to construct the virtual adult population have been described previously [31]. The paediatric demography (age, height, weight, BSA [body surface area]), developmental physiology (tissue volume, tissue blood flow, renal function, gastrointestinal tract anatomy) and biochemistry (albumin, CYP ontogeny) are integrated. The algorithms describing these developmental changes are described in detail elsewhere [32–36]. To account for the ontogeny of UGTs– specifically UGT1A1, UGT1A3, UGT2B4, UGT2B15, and UGT2B17 - the additional HLM clearance of ODV was adjusted based on literature-reported UGT levels at birth when simulating drug exposure in neonates (< 1 month old). The average activity of these UGT isoforms at birth is approximately 60% of the adult level; UGT1A1 and UGT2B17 show the lowest activity at birth, which is around 30% of the adult level [37, 38].

### Simulations

Simulations were performed using 10 trials of the number of subjects in the corresponding clinical studies. For individual PK data concerning lactating mothers and their infants, 100 trials per each subject were simulated. Where available, demographic information and dose regimens were aligned with those of corresponding clinical studies. When individual age data were unavailable, the reported age range was used. When sampling times are unspecified in the clinical study, steady-state concentrations of venlafaxine and ODV were simulated following multiple oral doses of venlafaxine for 7 days.

Venlafaxine and ODV are excreted into breast milk with observed mean (CV) of M/P AUC ratios of 2.78 (20%) and 2.82 (12%), respectively [12, 22]. These values were incorporated into the PBPK model along with the simulated average plasma concentration of venlafaxine and ODV to estimate the infant daily dose (IDD) using Eq. 1.

1 $$\eqalign{& {\rm{ IDD }}({\rm{mg}}/{\rm{kg}}/{\rm{ day }}) \cr & = {\rm{ Caverage }}({\rm{mg}}/{\rm{l}})\quad *{\rm{ daily milk intake }}(1/{\rm{kg}}/{\rm{ day }}) \cr} $$

The daily milk intake was assumed to be 150 mL/kg/day for exclusively breastfed infants. In cases where infants also received supplementary formula milk or solid food [12], breastmilk intake was assumed to be halved (75 mL/kg/day).

The relative infant daily dose (RIDD) was calculated using Eq. 2 where IDD represents the combined IDD values of both active moieties.

2 $$\eqalign{& {\rm{RIDD}}(\% ){\rm{ = }} \cr & {\rm{IDD }}({\rm{mg/kg/ day }}){\rm{/mother venlafaxine dose }}({\rm{mg/kg/ day }}) \cr} $$

Further, sensitivity analyses were performed to assess the impact of variability in milk composition (fat content and pH) on the M/P ratio using the algorithms published previously [39].

To simulate drug exposures in breast-fed infants, the estimated IDDs for venlafaxine and ODV were divided based on feeding frequency. For infants (less than 1 month old), IDDs were divided into 12 feeds every 2 h. For infants older than 1 month, IDDs were divided into 6 feeds and administered every 4 h. Simulated plasma ODV levels in infants formed by the metabolic conversion of venlafaxine and direct ODV intake from milk were added together to represent ODV levels in infants. The time-varying physiology features were also used when simulating drug exposures in infants [34].

Furthermore, to investigate the effects of various parameters to the observed variability, we simulated the exposures of venlafaxine and ODV in infants from birth to 1 year old, considering composite maternal/infant CYP2D6/CYP2C9/CYP2C19 polymorphisms, infant age-related physiological and biochemical maturations, as well as natural changes in feeding patterns and infant food consumption. The estimated average and maximum IDDs for both venlafaxine and ODV, derived from mothers who are EMs of CYP2D6/CYP2C9/CYP2C19 (All EMs), CYP2D6 PM, and PMs of CYP2D6/CYP2C9/CYP2C19 (all PMs), were administered to infants carrying the same CYP phenotypes based on the feeding frequencies corresponding to four age groups (less than 1 week, 1 week to 1 month, 1 to 6 months, 6 to 12 months). To simulate the likely real-world conditions, the average IDDs were used; for infants aged less than one week, 60% of UGT activity was assumed, reflecting the average activity at birth of UGT1A1, UGT1A3, UGT2B4, UGT2B15, and UGT2B17. Moreover, as food consumption typically increases in infants aged 6 months, with potentially reduced milk intake, we halved the IDD in this group (> 6 months). When investigating the worst-case scenarios, maximum IDDs were used without milk intake reduction in the older group, and a 30% UGT activity level was assumed for the youngest group, representing the lowest birth activity among these UGT isoforms (UGT1A1 and UGT2B17).

## Results

### Prediction of venlafaxine and ODV exposures in healthy adults with CYP2D6 genetic polymorphisms

**Fig. 3:**
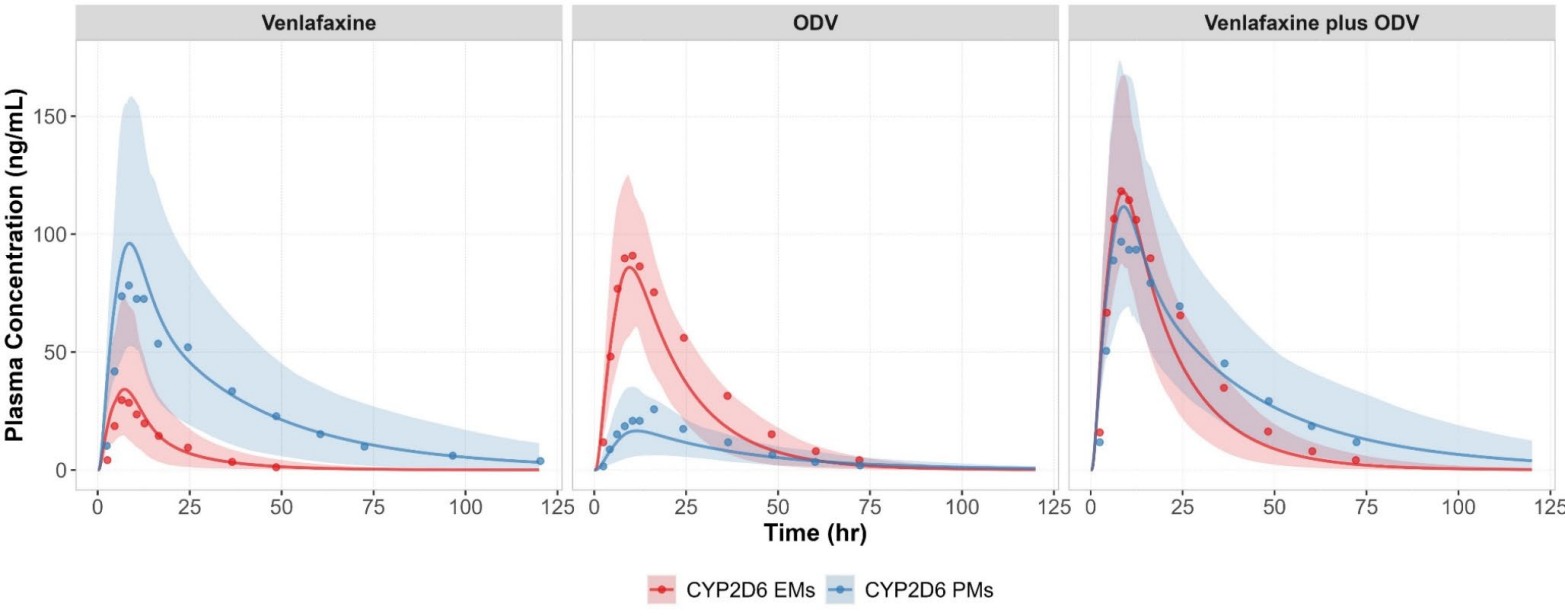
Simulated vs. observed plasma concentration-time profiles of venlafaxine, ODV and venlafaxine plus ODV in healthy adult CYP2D6 EMs and PMs. Simulated (lines) and observed (data points [25]), mean plasma concentration-time profiles of venlafaxine, ODV and venlafaxine plus ODV following a single oral dose of 75 mg venlafaxine ER. The shaded areas represent the 5th to 95th percentiles of total virtual populations

Simulated mean plasma concentration-time profiles of venlafaxine in a virtual healthy population following a single IV or an oral dose of venlafaxine (IR or ER formulation) recovered the observed data reasonably well (Figure S1 and Fig. 3). For ODV, the simulated concentration-time profiles following a single IV dose of desvenlafaxine or an oral dose of venlafaxine (IR and ER formulations) were consistent with these observed in healthy subjects (Figure S1). The models were verified further against pharmacogenomic data involving CYP2D6 EMs and PMs. Notably, venlafaxine concentrations were higher and ODV exposure was lower in CYP2D6 PMs compared with CYP2D6 EMs (Fig. 3a-b). Both venlafaxine and ODV demonstrate similar antidepressant efficacy, thus evaluating the combined concentrations of active moieties (venlafaxine plus ODV) over time is crucial. Interestingly, the observed combined exposure of active moieties remained consistent between CYP2D6 PMs and CYP2D6 EMs which was successfully captured by the models (Fig. 3c).

### Prediction of venlafaxine and ODV concentration-time profiles in nursing mothers

**Fig. 4:**
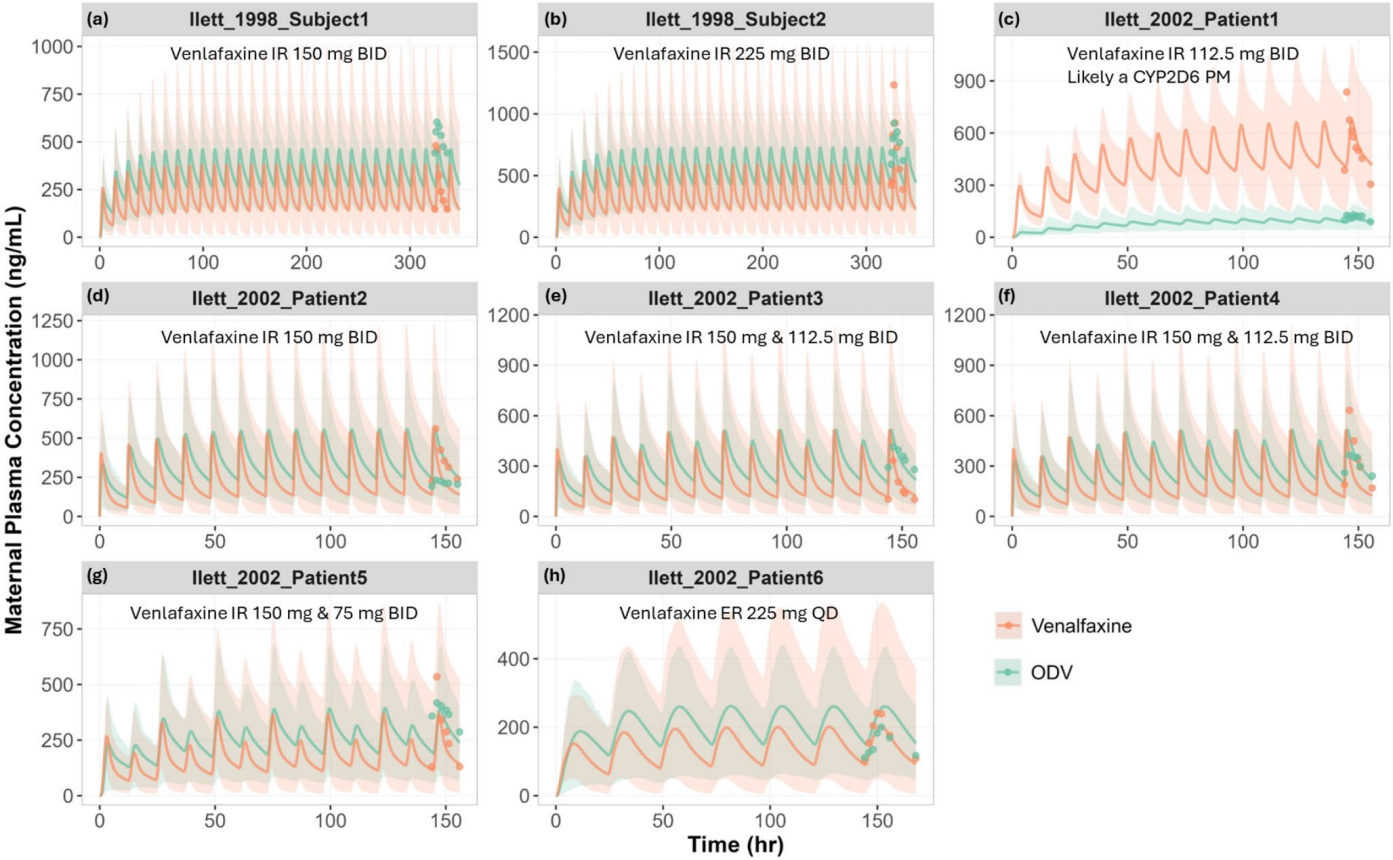
Simulated vs. observed maternal plasma concentration-time profiles of venlafaxine and ODV in lactating mothers. Simulated mean (lines) and observed individual (data points [12, 22]), maternal plasma concentration-time profiles of venlafaxine and ODV. The shaded areas represent the 5th to 95th percentiles of total virtual populations. Due to the unknown CYP2D6 genotypes/phenotypes in the clinical studies, the simulations used virtual populations representing the CYP2D6 phenotype frequencies of North European healthy adults. For subplot C, simulations assumed CYP2D6 PMs based on indications from Ilett et al. [12] that patient 1 is likely a CYP2D6 PM [12]

Concentration-time profiles of venlafaxine and ODV in lactating mothers who have taken daily doses of venlafaxine IR or ER formulations were available from two clinical studies [12, 22]. Predictions of the mean plasma concentration-time profiles of venlafaxine and ODV in the virtual female population consistent with the individual profiles reported in nursing mothers (Fig. 4).

### The impact of CYP2D6, CYP2C9 and CYP2C19 polymorphisms on IDD and RIDD for venlafaxine and ODV

Various scenarios were simulated to investigate the effects of CYP2D6, CYP2C9, and CYP2C19 polymorphisms on breast milk exposures of venlafaxine and ODV in mothers receiving the maximum daily dose of venlafaxine ER (225 mg QD) (Table 1). The estimated average IDD for the combined active moieties ranged from 0.131 mg/kg/day for all EMs to 0.259 mg/kg/day for all PMs with a maximum IDD of 0.666 mg/kg/day. Correspondingly, the estimated average RIDD ranged from 3.8% for all EMs to 7.6% for all PMs with a maximum RIDD of 17.6%.

**Table 1:**
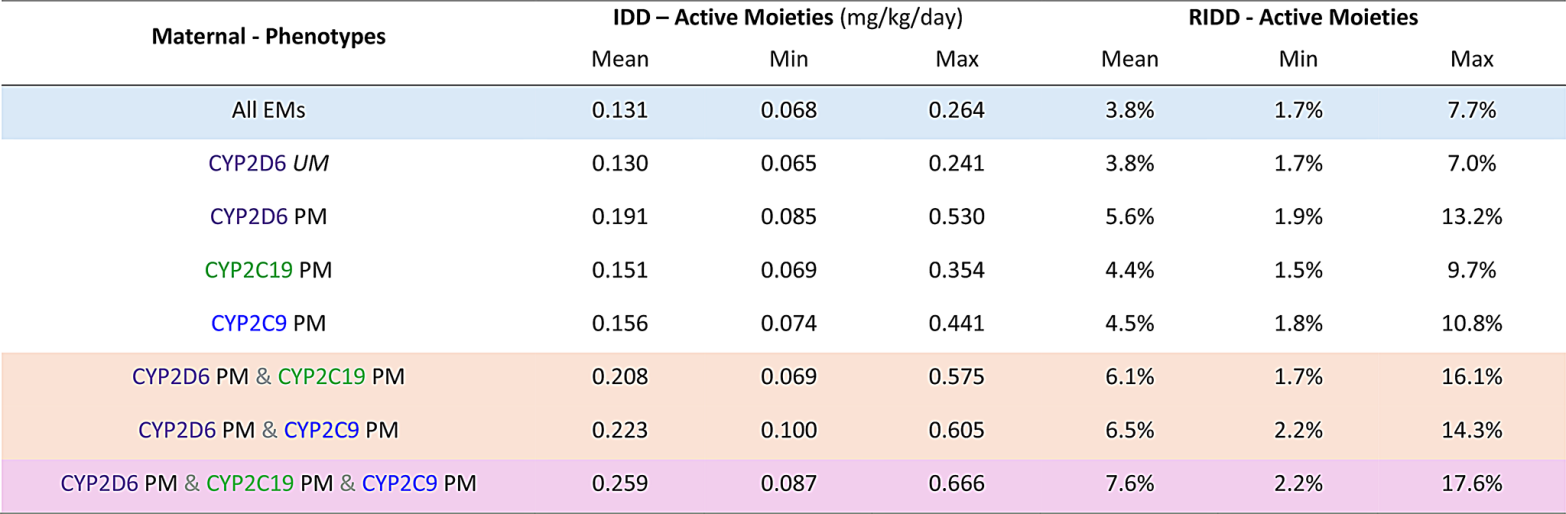
Estimated IDD and RIDD of combined active moieties (venlafaxine plus ODV) from 100 virtual lactating women aged 18–45 years old following 225 mg Venlafaxine ER once a day for each case

### The impact of physiological heterogeneity of breastmilk composition on venlafaxine and ODV exposures in breastmilk

The distribution of drugs into breastmilk is affected by the physicochemical properties of the drug (e.g., protein binding, lipophilicity and pKa) and the composition of the milk (e.g., aqueous, lipid, protein and pH) which exhibits physiological heterogeneity. For instance, the pH of breastmilk ranges from 7.0 to 7.4 [40, 41] and the fat content varies from 2.8 to 9.4% [42, 43]. To evaluate the impact of these dynamic changes in milk composition on the drug distribution in milk (M/P ratio), the theoretical M/P ratio for venlafaxine (2.44) and ODV (2.19) was predicted using the established algorithms based on the physicochemical properties and milk composition of 6.2% fat and pH = 7.0 [39]. These predicted ratios were reasonably consistent with the average M/P_AUC ratios from clinical studies, which are 2.78 for venlafaxine and 2.82 for ODV [12, 22]. Sensitivity analyses demonstrated that as milk fat content increases from 3 to 9%, the predicted M/P ratios for venlafaxine increase from 2.16 (at 3% fat) to 2.68 (at 9% fat), and for ODV, from 2.00 (at 3% fat) to 2.36 (at 9% fat). Additionally, the dynamic change in pH from 7.4 to 7.0 in milk resulted in a reduction of the M/P ratio from 0.98 (at pH 7.4) to 2.44 (at pH 7.0) for venlafaxine, and from 0.88 (at pH 7.4) to 2.19 (at pH 7.0) for ODV.

### Simulation of venlafaxine and ODV concentration-time profiles in breastfed infants

**Fig. 5:**
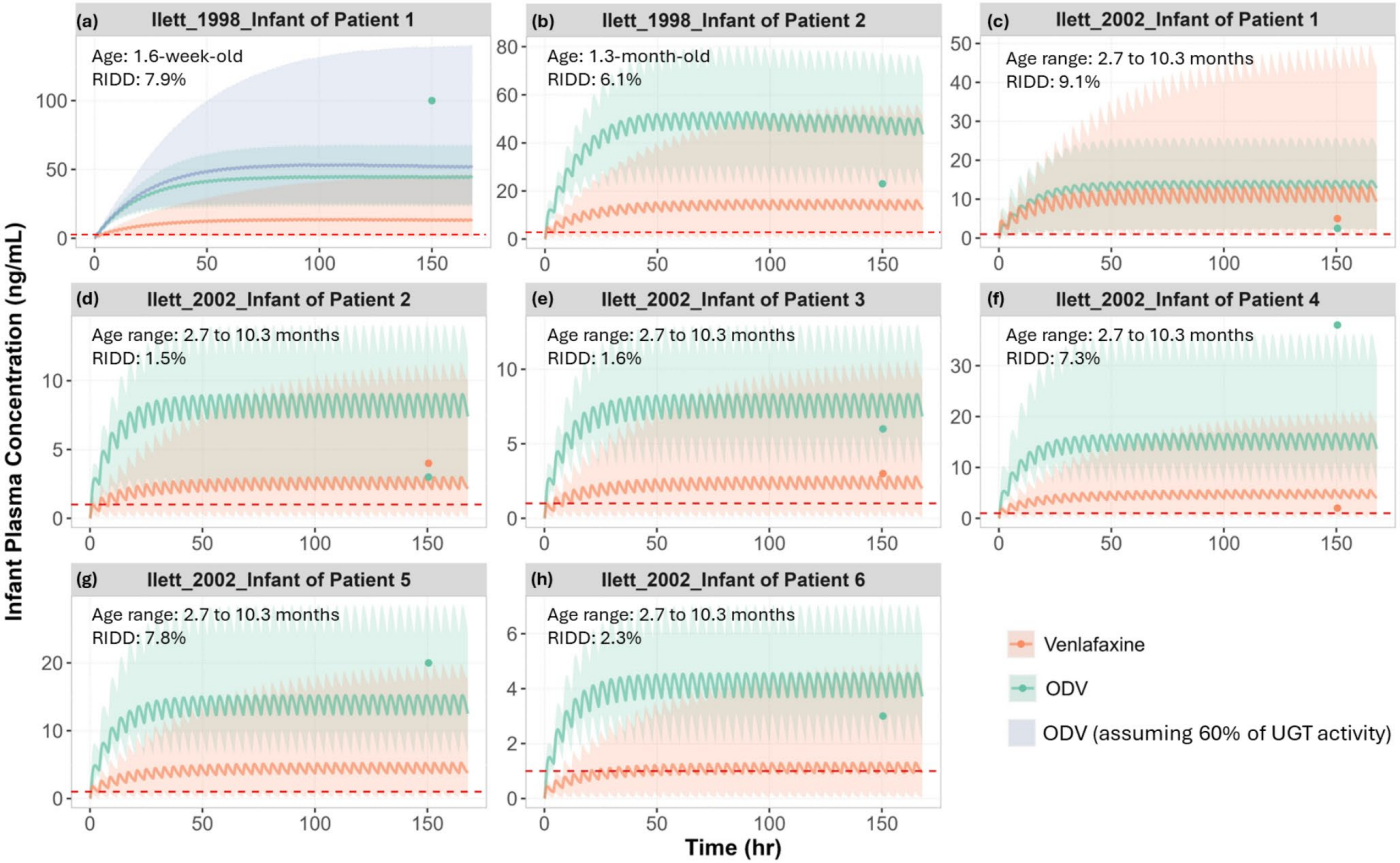
Simulated vs. observed plasma concentration-time profiles of venlafaxine and ODV in breastfed infants. Simulated mean plasma concentration-time profiles (lines) and observed individual (data points [12, 22]), concentrations of venlafaxine and ODV in breast-fed infants. The shaded areas represent the minimum to maximum of total virtual populations. The red dashed lines represent the detectable limit for venlafaxine and ODV in plasma. Venlafaxine plasma concentrations in infants of patients 1 and 2 from Ilett et al. [22] study and in infants of patients 5 and 6 from Ilett et al. [12] study are not detectable [12, 22]

Using the estimated IDDs for both active moieties from Table S3, we simulated the plasma concentration-time profiles for venlafaxine and ODV in infants from two clinical studies [12, 22]. Venlafaxine was not detectable in 4 out of 8 infant plasma samples. Specific blood sampling times for infants were provided in one clinical study [12]. Our simulations recovered 10 out of 12 clinically reported individual infant concentrations, as shown in Fig. 5. In Fig. 5a, the ODV level was underpredicted when assuming no ontogeny of UGT enzymes. By accounting for reduced UGT enzyme activity in newborns (60% of adult level), the simulations were able to capture the observed high ODV level in this 1.6-week-old subject. In Fig. 5f, the ODV level was slightly underpredicted. The rapid developmental changes in infants have significantly influences on the simulation results. However, the precise age was not provided; instead, the simulations used the reported age range.

**Fig. 6:**
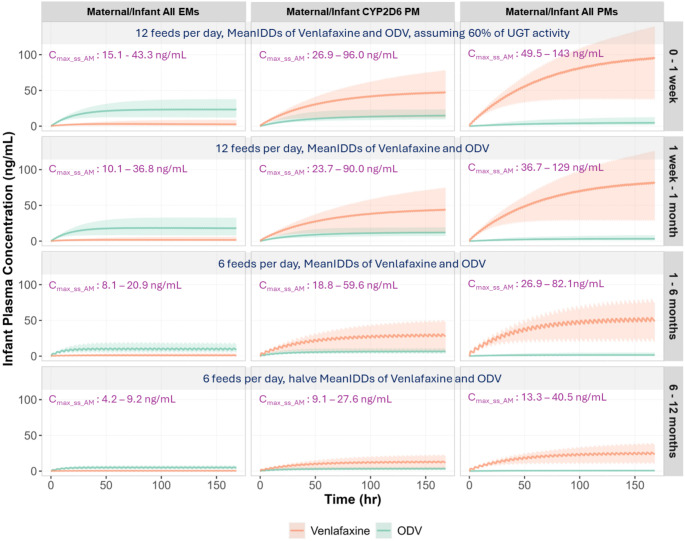
Simulated plasma concentration-time profiles of venlafaxine and ODV in breastfed infants associated with composite maternal/infant CYP polymorphisms. The estimated average IDDs (MeanIDDs) for both venlafaxine and ODV, derived from mothers who are EMs of CYP2D6, CYP2C9 and CYP2C19 (All EMs), PM of CYP2D6, and PMs of CYP2D6, CYP2C9 and CYP2C19 (All PMs). The doses were divided based on feeding frequencies and administered to infants with matched maternal phenotypes across four age groups (less than 1 week, 1 week to 1 month, 1 to 6 months, and 6 to 12 months). For infants under one week old, 60% of UGT activity was assumed, reflecting the average activity at birth of five UGT isoforms that contribute to ODV elimination. For infants older than 6 months, IDDs were halved to reflect the reduced milk intake due to food consumption. The lines represent the mean plasma concentration time profiles of venlafaxine and ODV. The shaded areas represent the minimum to maximum of total virtual populations per each age group. C_max_ss_AM_: maximum combined concentrations of Venlafaxine and ODV at steady-state

**Fig. 7:**
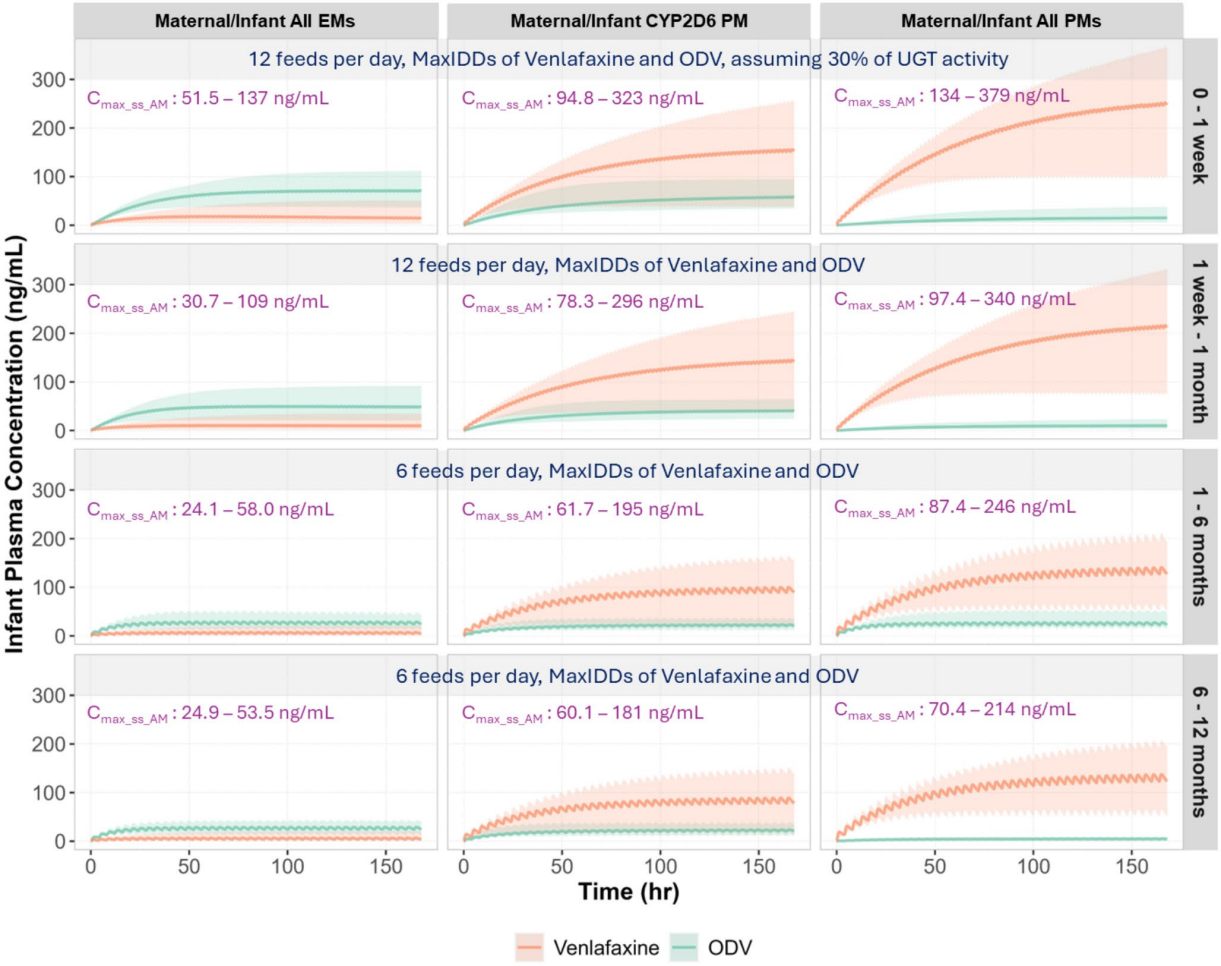
Simulated plasma concentration-time profiles of venlafaxine and ODV in breastfed infants associated with composite maternal/infant CYP polymorphisms considering worst-case scenarios. The estimated maximum IDDs (MaxIDDs) for both venlafaxine and ODV, derived from mothers who are EMs of CYP2D6, CYP2C9 and CYP2C19 (All EMs), PM of CYP2D6, and PMs of CYP2D6, CYP2C9 and CYP2C19 (All PMs). The doses were divided based on feeding frequencies and administered to infants with matched maternal phenotypes across four age groups (less than 1 week, 1 week to 1 month, 1 to 6 months, and 6 to 12 months). For infants under one week old, a 30% UGT activity level was assumed, representing the lowest birth activity among five UGT isoforms the contribute to ODV elimination. The lines represent the mean plasma concentration time profiles of venlafaxine and ODV. The shaded areas represent the minimum to maximum of total virtual populations per each age group. C_max_ss_AM_: maximum combined concentrations of Venlafaxine and ODV at steady-state

Furthermore, the exposures of venlafaxine and ODV in infants were simulated across four age groups (less than 1 week, 1 week to 1 month, 1 to 6 months, 6 to 12 months), accounting for CYP2D6/CYP2C9/CYP2C19 polymorphisms in both mothers and infants, enzyme ontogenies, renal function maturation, as well as natural changes in feeding patterns and infant food consumption. Figure 6 illustrates the likely real-world conditions for infants who received average IDDs, whereas Fig. 7 represents worst-case scenarios involving infants with significantly reduced UGT activity levels who received maximum IDDs. The exposure levels of venlafaxine and ODV were consistently lower in maternal-infant pairs classified as EMs of CYP2D6, CYP2C9 and CYP2C19 (All EMs), compared to those are PMs of CYP2D6, with the highest exposures predicted in PMs of CYP2D6, CYP2C9, and CYP2C19 (All PMs). With increasing age, both active moiety exposures decreased across the different polymorphism groups. In simulations performed under likely real-world conditions, the average maximum combined concentrations of active moieties (venlafaxine and ODV), C_max_ss_AM_, were 27.3, 21.0, 12.8, and 6.3 ng/mL in all EMs; 63.2, 57.0, 37.9, and 17.3 ng/mL in CYP2D6 PMs; 101, 85.8, 55.4, and 27.0 ng/mL in all PMs across four age groups from birth to 1 year old. In the worst-case scenarios, the average C_max_ss_AM_ values were 90.0, 61.8, 37.8 and 37.6 ng/mL in all EMs; 217, 188, 124, and 114 ng/mL in CYP2D6 PMs; 268, 226, 170, and 142 ng/mL in all PMs across four age groups. The minimum to maximum ranges across all tested scenarios are shown on Figs. 6 and 7.

## Discussion

A robust PBPK model describing the CYP2D6-, CYP2C19- and CYP2C9-mediated metabolism of venlafaxine and formed ODV was developed in this study. The relative contributions of the CYP enzymes were confirmed using clinical DDI studies (Table S2). A complex absorption model was able to capture plasma concentration time profiles of venlafaxine and formed ODV following administration of IR and ER formulations, both of which had been used across the various clinical studies. Consistent with observed data, simulations showed that the exposure of the combined active moieties (venlafaxine plus ODV) was similar for CYP2D6 EM and PM subjects [25]. Although clinical lactation data were available from several studies, including IR and ER formulations, CYP genotyping had not been conducted. After verifying that the PBPK model was able to capture maternal plasma concentrations of both venlafaxine and ODV, it was used to predict the impact of CYP polymorphisms on the IDD and RIDD following administration of the ER formulation. The estimated average IDD for the combined active moieties ranged from 0.131 mg/kg/day for all EMs to 0.259 mg/kg/day for CYP2D6 & CYP2C9 & CYP2C19 PMs with a maximum IDD of 0.666 mg/kg/day. Correspondingly, the estimated average RIDD ranged from 3.8% for all EMs to 7.6% for CYP2D6 & CYP2C9 & CYP2C19 PMs with a maximum RIDD of 17.6%. These estimates are consistent with clinical data where an average RIDD of 7.2% and a range of 3.0–13.3%, was reported across 3 studies [12, 13, 44]. Whilst the maximum predicted RIDD in nursing mothers who are PMs of CYP2D6, CYP2C9, and/or CYP2C19, exceeded the nominal 10% safety threshold, it is essential to note that the maximum predicted IDD remained substantially lower (15-fold) than the toxicity threshold (10 mg/kg) identified in a retrospective review of the dose-toxicity correlation for venlafaxine in patients aged 7 months to 19 years [45].

Even when clinical lactation data are available, there can be uncertainty associated with the IDD and RIDD estimates and whether they are in fact representative. These concerns in part relate to the timing of collection of the milk samples as the composition of the milk may change during the daily feeding schedule as well as during the postnatal period. The IDD is estimated from breast milk exposures, hence is dependent on the M/P ratio, which can be affected by the composition of the milk (fat content and pH). A sensitivity analyses indicated that the predicted M/P ratios of both venlafaxine and ODV were relatively insensitive to changes in fat content (fore *versus* hindmilk) but decreased about 2-fold with changes in pH (colostrum *versus* mature milk). Thus, the clinical IDD and RIDD estimates (based on M/P ratios of 2–3) are not likely to increase significantly by related changes in study design. Notably, simulations show that the RIDD was higher in mothers taking the same daily dose of venlafaxine in an IR formulation compared to an ER formulation. Although only venlafaxine ER (maximum daily dose of 225 mg) is available on the market now, it is important to be able to bridge across all available lactation data where various formulations may have been used.

Full concentration-time profiles of venlafaxine and ODV in lactating mothers who have taken daily doses of venlafaxine IR or ER formulations were available from two clinical studies [12, 22]. Predicted plasma profiles were deemed to be reasonably consistent with observed data from individual subjects (Fig. 4), with the combined exposures falling in the reported therapeutic range of 195 to 400 ng/mL [46]. Simulations in infants aged 1.3 to 10.3 months were able to capture 91% of observed exposures, albeit with limited samples (*n* = 11) (Fig. 5). In the neonate aged 1.6 weeks old, the relatively high concentration of ODV could only be recovered when an ontogeny of 60% was assumed for the UGT component of ODV elimination, which in effect is plausible.

Further simulations were conducted to address safety concerns about the use of venlafaxine during breastfeeding. As reported in the lactation database [9], concurrent side effects have rarely been reported in infants receiving venlafaxine and ODV through breastmilk. The therapeutic concentration range for the combined levels of venlafaxine and ODV is between 195 and 400 ng/mL [46]. Postmortem measurements in 22 cases associated with venlafaxine poisoning indicated venlafaxine levels ranging from 1,300 to 84,000 ng/mL, and combined venlafaxine and ODV levels ranging from 1,400 to 99,000 ng/mL [47–49]. Accounting for composite maternal/infant CYP2D6/CYP2C9/CYP2C19 polymorphisms, enzyme ontogenies, renal function, as well as natural changes in feeding patterns and food consumption, infant exposures of active moieties (venlafaxine plus ODV) were consistently higher in maternal/infant pairs classified as PMs of CYP2D6, CYP2C9 and CYP2C19 but remained < 145 ng/mL (Fig. 6). Not surprisingly, the worst-case scenario in terms of exposure (range: 134–379 ng/mL) occurred in neonates < 1 week old (all PMs) with only 30% UGT activity (Fig. 7). Furthermore, in these very young neonates, when the renal function was assumed to be zero, the average combined exposure was predicted to be 376 ng/mL (range: 208–559 ng/mL). The simulations confirm that both CYP polymorphisms and enzyme ontogenies affect the exposures of venlafaxine and ODV in infants and can explain the significantly variability observed in clinical studies.

The venlafaxine drug label does not provide enough information for healthcare professionals to decide whether this medication is safe during breastfeeding. The robust PBPK model presented here allowed us to investigate potential sources of variability associated with venlafaxine and ODV exposures in both nursing mothers and infants by considering complex factors, such as multiple genetic polymorphisms and time varying physiology. Even in extreme cases, the combined exposures of venlafaxine and ODV do not attain the levels reported to be associated with severe toxicity. The data presented here can be used to complement the existing clinical lactation data.

## Conclusion

Ethical issues around prescribing venlafaxine to breastfeeding mothers are linked to the potential safety risk to the infant without providing direct benefit. Expanding the existing knowledge base [9] especially relating to exposures of venlafaxine and ODV in breastfed neonates and infants can help clinicians and their patients make a more informed decision on the use of venlafaxine during lactation.

## Electronic supplementary material

Below is the link to the electronic supplementary material.

Supplementary Material 1

## Data Availability

No datasets were generated or analysed during the current study.
