# Supplementary material for: Advancing inclusive healthcare through PBPK modelling: predicting the impact of CYP genotypes and enzyme ontogenies on infant exposures of venlafaxine and its active metabolite O-desmethylvenlafaxine in lactation
Source: J Pharmacokinet Pharmacodyn. 2025 Apr 4;52(2):22. doi: 10.1007/s10928-025-09969-4 (PMC11971150; doi:10.1007/s10928-025-09969-4)
Supplement: Supplementary file 1 — Supplementary Material 1 [file 10928_2025_9969_MOESM1_ESM.docx]

**Method**

**Clinical Data**

PK data from healthy adult subjects were collated from 3 clinical studies to verify the model, including single intravenous (IV) dose of venlafaxine and desvenlafaxine (ODV), as well as a single oral dose of venlafaxine IR and ER formulations.

1. Study 1: Sixteen healthy men between 19 and 27 years of age, received a 30min-IV infusion of 10 mg venlafaxine or an oral dose of 50 mg venlafaxine IR (1).
2. Study 2: Healthy subjects aged 18 to 45 years were administered a 60min-IV infusion of 50 mg ODV (2). The number of subjects involved in the study are not provided.
3. Study 3: Fourteen healthy adult subjects aged 20-51 years received an oral dose of 75 mg venlafaxine ER. The study cohort includes 6 males and one female identified as CYP2D6 extensive metabolisers (EMs); and four males and three females identified as CYP2D6 poor metabolisers (PMs) (3).

Drug-drug interaction (DDI) studies were collected to verify each CYP component of the venlafaxine and ODV models, including the DDI studies with quinidine (CYP2D6 inhibitor), ketoconazole (CYP3A4, CYP2C9 and CYP2C19 inhibitor), and cimetidine (CYP2D6 inhibitor).

1. Quinidine study: Fourteen healthy men (eight CYP2D6 EMs, aged 22-30 years; six CYP2D6 PMs, aged 22-32 years) participated. Each subject received oral doses of 18.75 mg of venlafaxine hydrocholoride every 12 hr for 48 hr on two occasions: once alone and once during the concomitant administration of quinidine sulfate (100 mg every 12 h). The two study arms were performed 1 week apart (4).
2. Ketoconazole study: Twenty-one healthy volunteers (10 males and 11 females, 14 CYP2D6 EMs and 7 CYP2D6 PMs), aged 23-47 years participated in the study. In control arm, PMs and EMs received 25 mg and 50 mg single oral dose of venlafaxine, respectively. After 2-week wash-out period, subjects received 100 mg ketoconazole in the morning and evening on day 1. On day 2, subjects received ketoconazole (100 mg) and venlafaxine (25 mg for PMs and 50 mg for EMs) 1 h apart in the morning and 100 mg ketoconazole in the evening (5).
3. Cimetidine study: Eighteen healthy subjects (9 males and 9 females) aged 18-41 years participated received 25 mg of venlafaxine on day 1 and 50 mg of venlafaxine every 8 hours for the next 10 days. On the evening of day 6, each subject received an 800 mg oral dose of cimetidine at 10:00 PM, continuing with once daily administration of 800 mg through day 10 (6).

PK data from paediatric subjects are limited, therefore, therapeutic drug monitoring (TDM) data from one clinical study involving children and adolescents and one retrospective analysis that included adolescents were utilised to evaluate the model predictive performance in these groups.

1. Study 1: Twenty-one children aged 5-12 years (19% female) and adolescents aged 13-17 years (5.9% female) received venlafaxine for two weeks. Seven children and 7 adolescents were administered 0.5 mg/kg/day venlafaxine divided into two equal doses. Eight children and 5 adolescents were administered 1 mg/kg/day venlafaxine divided into two equal doses. Six children and 5 adolescents received 2 mg/kg/day venlafaxine divided into two equal doses. Plasma concentrations of venlafaxine and ODV were measured 7–10 hours post-morning dose on day 8 and day 15, with concentrations adjusted for dosage(7).
2. Retrospective analysis: Twenty-six subjects aged 12 to 17 years (77% female) received daily oral doses of venlafaxine ER ranging from 37.5 to 375 mg (median 131.25 mg). Blood samples were drawn at trough levels at steady state (8).

**Results**

Table S1. Input parameters of venlafaxine and ODV PBPK models

| **Venlafaxine** | | |
| --- | --- | --- |
| **Parameter** | **Value** | **Method/Reference** |
| Molecular weight (g/mol) | 277.4 | PubChem |
| log P | 2.9 | PubChem |
| Compound type | Monoprotic Base |  |
| pKa | 9.4 | (9) |
| B/P | 0.89 | Predicted |
| fu | 0.73 | (9) |
|  |  |  |
| Absorption Model | ADAM Model | |
| fu_gut_ | 1 | Assumed |
| Permeability Assay | Physiochemical |  |
| PSA (Å²) | 32.7 | PubChem |
| HBD | 1 |  |
| Formulation_1 | Immediate release (IR) | Optimised to recover the PK profile following an IR dose (10) |
|  | Dissolution profile  Weibull function alpha = 9.98, beta = 2.74 |  |
| Formulation_2 | Extended release (ER) | Optimised to recover the PK profile following an ER dose (11) |
|  | Dissolution profile  Weibull function alpha = 40.61, beta = 1.80 |  |
| Formulation_3 | Solution | To describe the absorption from breastmilk intake |
|  |  |  |
| Distribution Model | full PBPK Model |  |
| V_SS_ (L/kg) | 7.5 | Predicted based on Rodgers and Rowland method with an optimised Kp scalar to  recover the observed Vss (9) |
| Kp scalar | 0.65 |  |
|  |  |  |
| Enzyme | CYP2D6 |  |
| Pathway | O-demethylation |  |
| V_max_ (pmol/min/pmol) | 100.95 | Retrograde calculation – fm derived by in vitro data (12) |
| K_m_ (μM) | 23.3 | (12) |
|  |  |  |
| Enzyme | CYP2C19 |  |
| Pathway | O-demethylation |  |
| V_max_ (pmol/min/pmol) | 90.39 | Retrograde calculation – fm derived by in vitro data (12) |
| K_m_ (μM) | 293 | (12) |
|  |  |  |
| Enzyme | CYP2C9 |  |
| Pathway | O-demethylation |  |
| V_max_ (pmol/min/pmol) | 25.89 | Retrograde calculation – fm derived by in vitro data (12) |
| K_m_ (μM) | 3119 | (12) |
|  |  |  |
| Enzyme | CYP3A4 |  |
| Pathway | N-demethylation |  |
| V_max_ (pmol/min/pmol) | 5.90 | Retrograde calculation – fm derived by in vitro data (12) |
| K_m_ (μM) | 556 | (12) |
|  |  |  |
| Enzyme | CYP2C9 |  |
| Pathway | N-demethylation |  |
| V_max_ (pmol/min/pmol) | 72.39 | Retrograde calculation – fm derived by in vitro data (12) |
| K_m_ (μM) | 2250 | (12) |
|  |  |  |
| Enzyme | CYP2C19 |  |
| Pathway | N-demethylation |  |
| V_max_ (pmol/min/pmol) | 28.39 | Retrograde calculation – fm derived by in vitro data (12) |
| K_m_ (μM) | 398 | (12) |
|  |  |  |
| CL_R_ (L/h) | 3 | (9) |
|  |  |  |
| **ODV** | | |
| **Parameter** | **Value** | **Method/Reference** |
| Molecular weight (g/mol) | 263.37 | PubChem |
| log P | 2.6 | PubChem |
| Compound type | Monoprotic Base |  |
| pKa | 9.18 | (13) |
| B/P | 1.2255 | Predicted |
| fu | 0.7 | (2) |
|  |  |  |
| Absorption Model | ADAM Model |  |
| fu_gut_ | 1 | Assumed |
| Permeability Assay | Caco-2 |  |
| Permeability (10^-6^ cm/s) | 7.27 | (14) |
| Formulation | Solution | To describe the absorption from breastmilk intake |
|  |  |  |
| Distribution Model | full PBPK Model |  |
| V_SS_ (L/kg) | 3.4 | Predicted based on Rodgers and Rowland method with an optimised Kp scalar to  recover the observed Vss (2) |
| Kp scalar | 0.37 |  |
|  |  |  |
| CYP3A4 CL_int_ (μL/min/pmol) | 0.013 | Derived based on mass balance study (2). The UGT-mediated conjugation was assigned to additional Clint (HLM) and oxidative metabolism was assigned to CYP3A4. |
|  |  |  |
| Additional CL_int_ (HLM) (μL/min/mg protein) | 1.769 |  |
|  |  |  |
| CL_R_ (L/h) | 12.1 | (2) |
|  |  |  |

**Abbreviations:**

LogP: Log of the octanol:water partition coefficient; fu: fraction unbound in plasma; B/P: blood-to-plasma partition ratio; ADAM: Advanced Dissolution, Absorption and Metabolism; fu_gut_: unbound fraction in enterocyte; PSA: polar surface area; HBD: hydrogen bound donor; Vss: volume of distribution at steady state; Kp scalar: scalar applied to all predicted tissue to plasma partition coefficients; K_m_: Michaelis-Menten constant accounting for the binding in vitro system; V_max_: maximum rate of metabolite formation; CL_int_: *in vitro* intrinsic clearance; HLM: human liver microsomes; CL_R_: renal clearance;

**Prediction of DDIs between venlafaxine and CYP2D6, CYP3A4, CYP2C9 and CYP2C19 inhibitors**

Clinical DDI studies involving CYP2D6, CYP3A4, CYP2C9 and CYP2C19 inhibitors (quinidine, ketoconazole, and cimetidine) involving CYP2D6EMs and CYP2D6 PMs were utilised to verify each CYP component within the models. The predictions were all within 1.5-fold of the observed data. Collectively, 10 out of 12 predictions fell within 0.8- to 1.25-fold of the observed data (Table S2). During coadministration of quinidine (a CYP2D6 inhibitor), oral clearance of venlafaxine significantly decreased in CYP2D6 EMs but remained unchanged in CYP2D6 PMs, reflecting the inactive CYP2D6 enzyme in these individuals. In contrast, coadministration of ketoconazole (CYP3A4, CYP2C9 and CYP2C19 inhibitor), increased venlafaxine exposure more significantly in CYP2D6 PMs compared to EMs due to the increased fm of CYP3A4, CYP2C9 and CYP2C19 for venlafaxine in CYP2D6 PMs. Coadministration with ketoconazole had a more pronounced effect on increasing ODV exposure in CYP2D6 EMs than in PMs, because the formation of ODV was blocked by ketoconazole and the inactivity of CYP2D6 in CYP2D6 PMs. Coadministration of cimetidine (a CYP2D6 inhibitor) increased the exposure of venlafaxine and reduced the formation of ODV. All these clinical findings were successfully captured by the models.

Table S2. Observed and predicted men CL, Cmax and AUC ratios for interaction with venlafaxine following coadministration of quinidine, ketoconazole or cimetidine.

|  |  | Venlafaxine | | | ODV | | |
| --- | --- | --- | --- | --- | --- | --- | --- |
|  |  | Observed | Predicted  (trial range) | P/O | Observed | Predicted  (trial range) | P/O |
| Quinidine | CL ratio (CYP2D6 EMs) | 0.17 | 0.25  (0.21, 0.35) | 1.47 | NA | NA | NA |
|  | CL ratio (CYP2D6 PMs) | 1.00 | 0.99  (0.99, 1.00) | 0.99 | NA | NA | NA |
| Ketoconazole* | AUC ratio (CYP2D6 EMs) | 1.21 | 1.15  (1.10, 1.19) | 0.95 | 1.22 | 1.33  (1.28, 1.41) | 1.09 |
|  | Cmax ratio (CYP2D6 EMs) | 1.20 | 1.10  (1.07, 1.12) | 0.92 | 1.21 | 1.12  (1.10, 1.15) | 0.92 |
|  | AUC ratio (CYP2D6 PMs) | 1.54 | 1.43  (1.33, 1.61) | 0.92 | 1.12 | 1.16  (1.09, 1.26) | 1.03 |
|  | Cmax ratio (CYP2D6 PMs) | 1.39 | 1.24  (1.18, 1.31) | 0.89 | 1.14 | 0.86  (0.81, 0.91) | 0.75 |
| Cimetidine | AUC ratio | 1.62 | 1.70  (1.55, 1.95) | 1.05 | NR | 0.88  (0.73, 0.81) | NA |
|  | Cmax ratio | 1.59 | 1.68  (1.55, 1.94) | 1.06 | NR | 0.77  (0.82, 0.90) | NA |

Predicted values show mean and trial range from the simulation trial matching the clinical study design.

CL: oral clearance; NR: not reported; N.A.: not applicable; *CYP2C19 and CYP2C9 competitive inhibition Ki values of 0.183 and 0.08 obtained from in vitro studies (15, 16) were incorporated into ketoconazole file.

**Figure S1. Simulated vs observed plasma concentration-time profiles of venlafaxine and ODV in healthy adults.** Simulated (lines) and observed (data points, (1, 2)) mean plasma concentration-time profiles of venlafaxine and ODV. The shaded areas represent the 5^th^ to 95^th^ percentiles of total virtual populations.


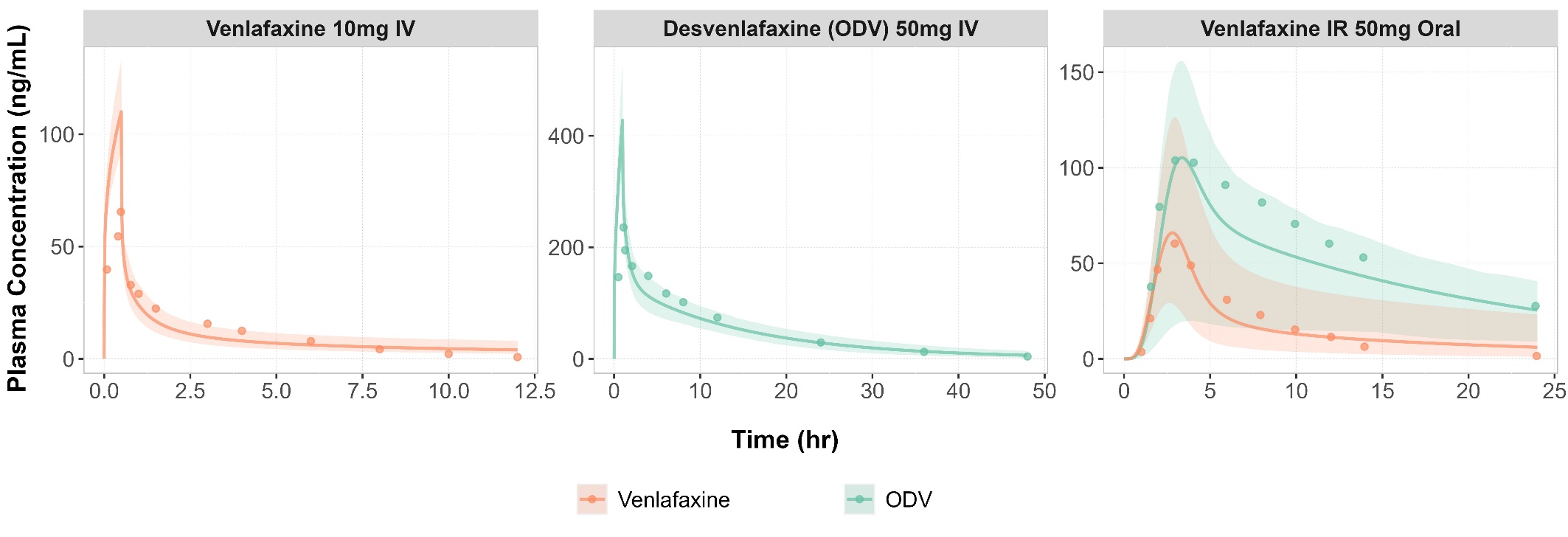


**Prediction of venlafaxine and ODV concentrations in paediatrics.**

PK profiles of venlafaxine and ODV concentration in paediatrics are scarce. Findling et al reported median and range of measurable venlafaxine and ODV concentrations at 7-10 h post dose on day 8 and 15 following a BID dosing of venlafaxine IR in 21 children (5-12 years old) and 17 adolescents (13-17 years old) for 2 weeks (7). The predicted median and range (minimum to maximum) of plasma concentrations of venlafaxine and ODV recovered the clinically observations reasonably well (Figure S2). Moreover, TPM data collected from the retrospective analysis in 26 adolescents who received QD dose of venlafaxine ER (8) showed that the simulated ranges well captured the clinically observed variability in trough concentrations (Figure S2).

**Figure S2.** **Simulated vs observed plasma concentrations of venlafaxine and ODV in children and adolescents.** Simulated (lines) median plasma concentration-time profiles and observed (data points (7, 8) median plasma concentrations of venlafaxine (a-c) and ODV (d-f) in children aged 5-12 years and adolescents aged 12-17 years. The error bars represent the observed data range. The shaded areas represent the minimum to maximum of total virtual populations.


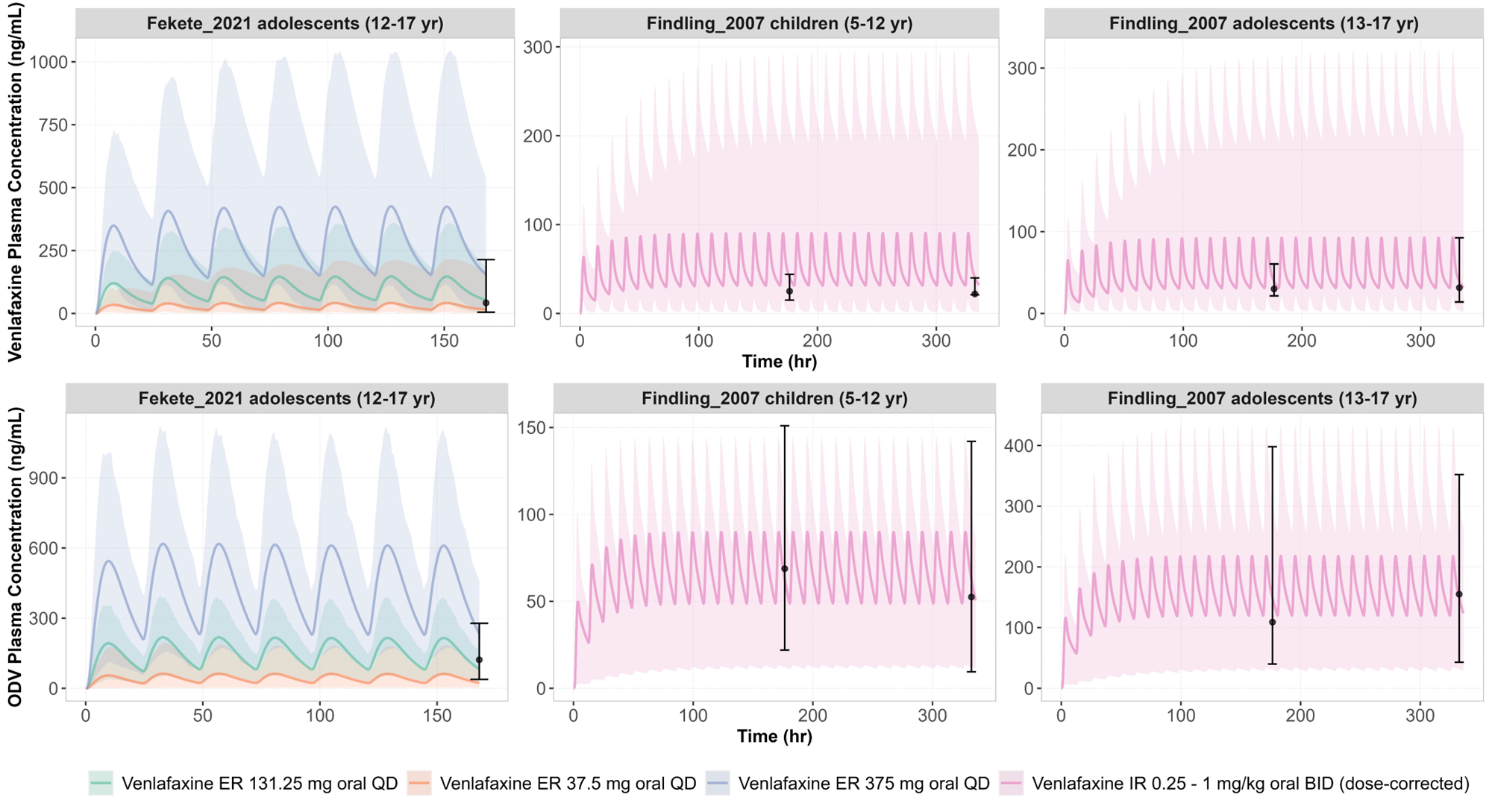


Table S3. Estimated average IDD from clinical studies

| **Study** | **Mother** | **Venlafaxine**  **formulation** | **Venlafaxine Dose** | | **IDD (mg/kg/day)** | | | **IDD (mg/kg/feed), 6 feeds/day** | | | **RIDD** |
| --- | --- | --- | --- | --- | --- | --- | --- | --- | --- | --- | --- |
|  |  |  | mg/day | mg/kg/day | Venlafaxine | ODV | Venlafaxine | | | ODV |  |
| Ilett 1998 (17) | 1 | IR | 300 | 4.11 | 0.133 | 0.181 | 0.0222 | | 0.0302 | | 7.9% |
|  | 2 | IR | 450 | 8.18 | 0.206 | 0.281 | 0.0344 | | 0.0468 | | 6.1% |
| Ilett 2002 (18) | 1^#^ | IR | 225 | 2.67 | 0.205 | 0.037 | 0.0342 | | 0.0062 | | 9.1% |
|  | 2 | IR | 300 | 3.56 | 0.047* | 0.006* | 0.0078* | | 0.0120* | | 1.5% |
|  | 3 | IR | 262.5 | 3.11 | 0.044* | 0.006* | 0.0073* | | 0.0110* | | 1.6% |
|  | 4 | IR | 262.5 | 3.11 | 0.087 | 0.132 | 0.0145 | | 0.0220 | | 7.3% |
|  | 5 | IR | 225 | 2.67 | 0.082 | 0.120 | 0.0137 | | 0.0199 | | 7.8% |
|  | 6 | ER | 225 | 2.67 | 0.021* | 0.038* | 0.0034* | | 0.0064* | | 2.3% |

^#^: mother 1 is likely a CYP2D6 PM according to the clinical study (18). *The estimation was based on daily milk intake of 75 mL/kg/day due to supplementary feeding of solid food or formula milk.

**References**

(1) Patat, A. *et al.* Absolute bioavailability and electroencephalographic effects of conventional and extended-release formulations of venlafaxine in healthy subjects. *J Clin Pharmacol* **38**, 256-67 (1998).

(2) *Desvenlafaxine. NDA: 21-992.Clinical Pharmacology and Biopharmaceutics Review*. <[www.accessdata.fda.gov/drugsatfda_docs/nda/2008/021992s000_ClinPharmR_P4.pdf](file:///C:\Users\kryeo\Downloads\www.accessdata.fda.gov\drugsatfda_docs\nda\2008\021992s000_ClinPharmR_P4.pdf)>. Accessed August 22 2023.

(3) Nichols, A.I., Focht, K., Jiang, Q., Preskorn, S.H. & Kane, C.P. Pharmacokinetics of venlafaxine extended release 75 mg and desvenlafaxine 50 mg in healthy CYP2D6 extensive and poor metabolizers: a randomized, open-label, two-period, parallel-group, crossover study. *Clin Drug Investig* **31**, 155-67 (2011).

(4) Lessard, E., Yessine, M.A., Hamelin, B.A., O'Hara, G., LeBlanc, J. & Turgeon, J. Influence of CYP2D6 activity on the disposition and cardiovascular toxicity of the antidepressant agent venlafaxine in humans. *Pharmacogenetics* **9**, 435-43 (1999).

(5) Lindh, J.D., Annas, A., Meurling, L., Dahl, M.L. & A, A.L.-S. Effect of ketoconazole on venlafaxine plasma concentrations in extensive and poor metabolisers of debrisoquine. *Eur J Clin Pharmacol* **59**, 401-6 (2003).

(6) Troy, S.M., Rudolph, R., Mayersohn, M. & Chiang, S.T. The influence of cimetidine on the disposition kinetics of the antidepressant venlafaxine. *J Clin Pharmacol* **38**, 467-74 (1998).

(7) Findling, R.L. *et al.* Venlafaxine in the treatment of children and adolescents with attention-deficit/hyperactivity disorder. *J Child Adolesc Psychopharmacol* **17**, 433-45 (2007).

(8) Fekete, S. *et al.* Dose-Corrected Serum Concentrations and Metabolite to Parent Compound Ratios of Venlafaxine and Risperidone from Childhood to Old Age. *Pharmacopsychiatry* **54**, 117-25 (2021).

(9) *Effexor XR (venlafaxine extended-release) capsules. Drug lable. FDA.* <<https://www.accessdata.fda.gov/drugsatfda_docs/label/2017/020699s107lbl.pdf>>. Accessed August 11 2023.

(10) Hynninen, V.V., Olkkola, K.T., Bertilsson, L., Kurkinen, K., Neuvonen, P.J. & Laine, K. Effect of terbinafine and voriconazole on the pharmacokinetics of the antidepressant venlafaxine. *Clin Pharmacol Ther* **83**, 342-8 (2008).

(11) Godoy, A.L., Rocha, A., da Silva Souza, C. & Lanchote, V.L. Pharmacokinetics of venlafaxine enantiomers and their metabolites in psoriasis patients. *J Clin Pharmacol* **56**, 567-75 (2016).

(12) Fogelman, S.M. *et al.* O- and N-demethylation of venlafaxine in vitro by human liver microsomes and by microsomes from cDNA-transfected cells: effect of metabolic inhibitors and SSRI antidepressants. *Neuropsychopharmacology* **20**, 480-90 (1999).

(13) Franek, F., Jarlfors, A., Larsen, F., Holm, P. & Steffansen, B. In vitro solubility, dissolution and permeability studies combined with semi-mechanistic modeling to investigate the intestinal absorption of desvenlafaxine from an immediate- and extended release formulation. *Eur J Pharm Sci* **77**, 303-13 (2015).

(14) Oganesian, A. *et al.* Desvenlafaxine and venlafaxine exert minimal in vitro inhibition of human cytochrome P450 and P-glycoprotein activities. *Psychopharmacol Bull* **42**, 47-63 (2009).

(15) Foti, R.S. & Wahlstrom, J.L. CYP2C19 inhibition: the impact of substrate probe selection on in vitro inhibition profiles. *Drug Metab Dispos* **36**, 523-8 (2008).

(16) Kumar, V., Wahlstrom, J.L., Rock, D.A., Warren, C.J., Gorman, L.A. & Tracy, T.S. CYP2C9 inhibition: impact of probe selection and pharmacogenetics on in vitro inhibition profiles. *Drug Metab Dispos* **34**, 1966-75 (2006).

(17) Ilett, K.F. *et al.* Distribution and excretion of venlafaxine and O-desmethylvenlafaxine in human milk. *Br J Clin Pharmacol* **45**, 459-62 (1998).

(18) Ilett, K.F., Kristensen, J.H., Hackett, L.P., Paech, M., Kohan, R. & Rampono, J. Distribution of venlafaxine and its O-desmethyl metabolite in human milk and their effects in breastfed infants. *Br J Clin Pharmacol* **53**, 17-22 (2002).
